# Computer-aided designing of immunosuppressive peptides based on IL-10 inducing potential

**DOI:** 10.1038/srep42851

**Published:** 2017-02-17

**Authors:** Gandharva Nagpal, Salman Sadullah Usmani, Sandeep Kumar Dhanda, Harpreet Kaur, Sandeep Singh, Meenu Sharma, Gajendra P. S. Raghava

**Affiliations:** 1 CSIR-Institute of Microbial Technology, Sector 39A, Chandigarh, - 160036 India

## Abstract

In the past, numerous methods have been developed to predict MHC class II binders or T-helper epitopes for designing the epitope-based vaccines against pathogens. In contrast, limited attempts have been made to develop methods for predicting T-helper epitopes/peptides that can induce a specific type of cytokine. This paper describes a method, developed for predicting interleukin-10 (IL-10) inducing peptides, a cytokine responsible for suppressing the immune system. All models were trained and tested on experimentally validated 394 IL-10 inducing and 848 non-inducing peptides. It was observed that certain types of residues and motifs are more frequent in IL-10 inducing peptides than in non-inducing peptides. Based on this analysis, we developed composition-based models using various machine-learning techniques. Random Forest-based model achieved the maximum Matthews’s Correlation Coefficient (MCC) value of 0.59 with an accuracy of 81.24% developed using dipeptide composition. In order to facilitate the community, we developed a web server “IL-10pred”, standalone packages and a mobile app for designing IL-10 inducing peptides (http://crdd.osdd.net/raghava/IL-10pred/).

The tolerance mechanism of the immune system is well regulated and under surveillance. Yet, a prolonged or excessive immune response leads to auto-immunity that could be overcome by immunosuppression mediated by anti-inflammatory cytokines like IL-10^1^,^2^, IL-37^3^, IL-33^3^,^4^, IL-4^3^, IL-13^3^, IL-35^5^,^6^, TGF-β^3^,^7^. One of the well-known cytokines responsible for immunosuppression is IL-10^1^, which plays a critical role in preventing inflammatory responses, alleviating autoimmune pathologies^2^ and in prolonging graft survival^8^,^9^. Fiorentino *et al*.^10^ observed that the T helper 2 (T_h_2) cell clones inhibit interferon-γ (IFN-γ) synthesis in T helper 1 (T_h_1) cell clones by releasing a cytokine later named as Interleukin-10 (IL-10)^10^. Initially, IL-10 was considered as a T_h_2-type cytokine^10^ but several studies conducted during the last two decades, concluded that IL-10 is a broadly expressed cytokine^11^,^12^,^13^,^14^,^15^,^16^.

Almost all the cells of the immune system express IL-10 including macrophages^17^, dendritic cells (DCs)^3^, neutrophils^3^, B cells^18^,^19^, T cells and mast cells^20^. Activation of the T-cell receptor and the signal transducer and activator (STAT) of transcription pathway causes the differentiation of native CD4^+^ T cells into T_h_ cells^15^. Under certain conditions and presence of other cytokines, T_h_1^10^,^21^,^22^,^23^, T_h_2^10^, Th3^24^, Th9^25^ and T_h_17^26^ express IL-10^16^,^27^,^28^. ERK pathway also plays an important role in regulating the production of IL-10 in dendritic cells and macrophages^23^. CD8^+^ T-cells also express IL-10 upon T-cell receptor (TCR) activation and interaction with activated plasmacytoid dendritic cells^29^. Auto-antigens, TLR-4^15^, TLR-9^30^ and vitamin D3^31^ can stimulate B-cells to produce IL-10^15^,^18^. Similarly, damaged skin or TLR-4 activation induces the expression of IL-10 in mast cells^15^,^32^ (Fig. 1). This cytokine IL-10 inhibits CD28 signaling pathway and arrests the T-cells in the anergy^1^. It also regulates antibody isotypes, inhibits dendritic cell maturation and reduces the release of inflammatory cytokines by the mast cells^1^,^7^ (Fig. 2).

In the last three decades, a number of methods have been developed for predicting T-cell epitopes^33^. These methods can be broadly classified into two categories; direct and indirect methods. The indirect methods (e.g., Pclevage^34^, NetChop^35^, Propred^36^, ProPred1^37^, TAPPred^38^) predict only one component of the pathway of T-cell recognition; for example, ProPred-I predicts MHC class-1 binders rather than T-cell epitopes. CTLPred is an example of the methods that directly predict Cytotoxic T-lymphocyte (CTL) epitope rather than MHC binders^39^. These methods directly or indirectly predict T-cell epitopes but they do not provide information on the release of cytokines. Recently our group has taken the initiative to develop cytokine-specific prediction methods (e.g., IFNepitope^40^, IL4Pred^41^). To the best of author’s knowledge, there is no method for the prediction of IL-10 inducing epitopes. This study is an attempt to develop computational models for predicting peptides that can induce cytokine IL-10 production.

## Results

In this study, we used 394 MHC class-II binders, which have the ability to induce cytokine IL-10, as positive instances. On the other hand, we used 848 MHC class-II binders, which do not have the ability to induce cytokine IL-10, as negative examples. Thus, our dataset consisted of 394 IL-10 inducing and 848 non-inducing peptides or epitopes. We performed all the analysis on this dataset to understand the preference of residues and motifs in IL-10 inducing peptides. It was observed that all the peptides contained at least 8 residues. The maximum length of the peptides was observed to be 42 in the positive set and 27 in the negative set. Based on the analysis, we developed prediction models wherein all models were trained and tested on this dataset.

### Positional Conservation Analysis

**Figure 3:**
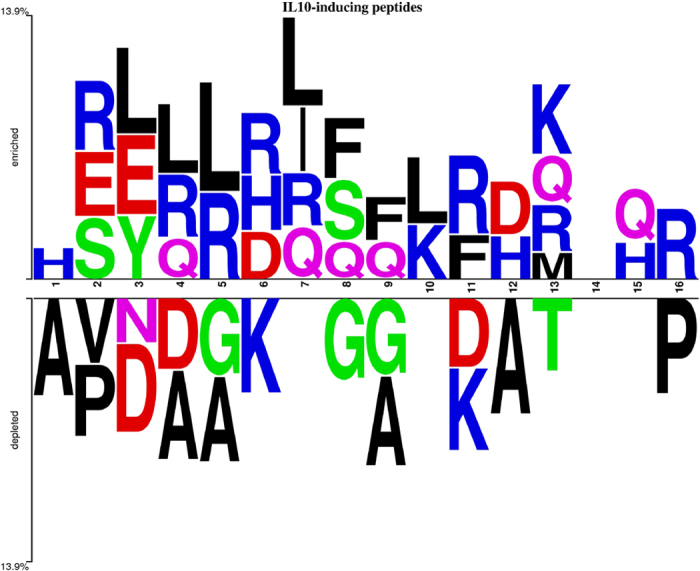
Visualization of residues conserved in IL-10 inducing and non-inducing peptides using two-sample logo.

In order to understand the preference of specific residues at certain positions, we generated a two-sample logo (TSL) for the positive and negative peptides (Fig. 3). In a TSL, the height of the amino acid symbol is indicative of its relative abundance. The number of terminal residues was selected on the basis of the minimum peptide length in the dataset and is not associated with any biological function. It has been observed that R is highly preferred at position 2^nd^, 4^th^, 5^th^, 6^th^, 7^th^, 11^th^, 13^th^, and 16^th^ in IL-10 inducing peptides. Similarly, L is more dominant at position 3^rd^, 4^th^, 5^th^, 7^th^ and 10^th^ in IL-10 inducing peptides. On the other hand, the residue A was found to be predominant in non-IL-10 inducing peptides at 1^st^, 4^th^, 5^th^, 9^th^ and 12^th^ position.

### Compositional Analysis

**Figure 4:**
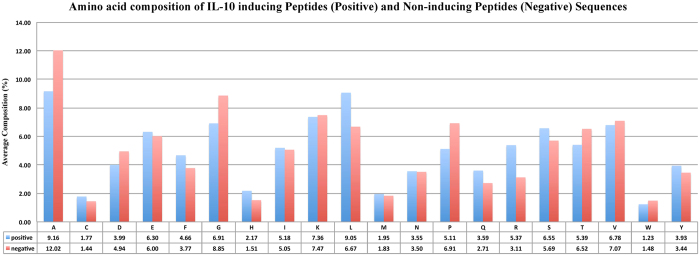
Bar graph shows average amino acid composition of IL-10 inducing and non-inducing peptides.

The Amino Acid Composition (AAC) was computed for IL-10 inducing and non-inducing peptides; the average composition is shown in the bar plot (Fig. 4). As shown in Fig. 4, certain residues (like A, G and P) have a higher average composition in non-inducing or negative peptides than in positive peptides. In contrast, the residues L and R are more abundant in IL-10 inducing peptides.

### Motif based analysis

In the present work, we used MERCI program^42^ for searching motifs occurring exclusively in IL-10 inducing peptides but not found in non-inducing peptides. Similarly, we searched motifs exclusively found in IL-10 non-inducing peptides. As shown in Table 1, the motifs found in IL-10 inducing peptides are rich in R, K and L while the exclusive motifs found in non-inducing peptides are dominated by residues A, G and P. Notably, the residue V is prevalent in the exclusive motifs of both the negative as well as the positive sets.

### Support Vector Machine-based models

We developed prediction models using Support Vector Machine (SVM) for discriminating IL-10 inducing and non-inducing sequences. Various sequence-based features of the peptides were used as input for developing SVM-based prediction models. Amongst the amino acid composition (AAC) models, we obtained the highest accuracy of 72.30% with Matthews’s correlation coefficient (MCC) value 0.41 (Table 2). The performance of our prediction model improved significantly using the dipeptide composition (DPC) as input feature instead of the AAC. As shown in Table 2, SVM model achieves maximum accuracy 78.42% with MCC value 0.55 using dipeptide composition. In addition, we developed models using terminal composition of peptides^43^. Since the minimum length of the peptides in our dataset is 8, we extracted 8 residues from N-terminus and developed the model called NT8 using AAC and DPC; these models achieved the maximum accuracy values of 63.45% and 66.75% respectively as shown in Table 2. Further, the models developed using the binary profiles of amino acids in the peptides, attained the accuracy of 67.15% with MCC of 0.31 for NT8. In the case of the CT8 models (involving the input features of terminal 8 residues of the C-terminus of the peptides), the AAC and DPC features obtained the accuracies 63.85% and 65.22% respectively. The binary model for the CT8 showed an accuracy of 62.88%. Additionally, we concatenated 8 residue sequences each at the N and C terminals to develop the NT8CT8 model, where a slight increase in the performance was observed as compared to models developed separately for NT8 or CT8 terminal input features. The maximum MCC value obtained here was 0.54 with DPC input vector.

In this study, various models were also developed using split composition^44^, where the peptide sequence is split into two equal parts. The compositions of the two parts are used as the input features for developing models. These models achieved the accuracy values of 73.67% and 72.71% for split-AAC and split-DPC respectively. In order to reduce the noise in models, we removed less significant or insignificant features. The CfSubSetEval algorithm of WEKA was used for selecting important features from AAC and DPC, with16 and 57 features respectively being selected by the algorithm, as enlisted in Table S1. These selected features were further used for developing SVM-based models. The models developed on the selected features performed less than the models based on all features taken together (Table S2).

### Models using WEKA classifiers

We have also used the WEKA suite, which is a collection of various machine-learning algorithms. Out of many algorithms available in WEKA, we have employed four classifiers IBK, SMO, J48 and Random Forest. IBK (a K-nearest neighbors classifier) based model using AAC achieved the maximum accuracy 73.51% with MCC 0.44. Sequential minimal optimization (SMO) reached the maximum accuracy of 74.40%, 78.66% and MCC of 0.37, 0.49 using AAC and DPC respectively. J48 is a tree-based machine learning classifier in the WEKA package that attained the accuracy values of 68.28% and 67.15% for AAC and DPC respectively. Notably, the models based on Random Forest achieved the maximum accuracy value of 80.11% with MCC 0.58 for AAC. In the case of DPC, a Random Forest-based model achieved an accuracy of 81.24% with MCC value of 0.59 (Table 3).

We also developed models based on IBK, SMO and J48 classifier using split-AAC and achieved the maximum accuracy of ~71%. In the case of split-DPC, the performances achieved using these classifiers were comparable to split-AAC. Models based on Random Forest performed better than other classifiers and attained the maximum MCC 0.50 using split-AAC (Table S3). We also developed Random Forest-based models using 16 selected features from AAC and achieved the maximum MCC of 0.55 (Table 4). Similarly, we developed models based on WEKA classifiers using selected features from DPC.

### External Validation

The external validation technique is one of the most rigorous techniques commonly used to evaluate the realistic performance of a model. In this technique, the performance of a model is evaluated on a dataset not used for its training or testing; this dataset is called independent or validation dataset. In order to evaluate the performance of our models we extracted 66 IL-10-inducing peptides, recently added in IEDB. These peptides are not available in our original dataset used for building models. The best SVM model correctly predicted 45 out of 66 peptides newly included by IEDB as IL-10-inducing MHC-II binding peptides. The Random Forest model with the best performance found in our study correctly predicted 55 out of these 66 peptides. This demonstrates that our models are rigorous and their performance is reasonably good on the independent dataset.

### Classification of IL-10-inducing and MHC II non-binding peptides

The prediction models described above are suitable to classify IL-10 inducing and non-inducing peptides in MHC II binding peptides. This means the user cannot use these models to predict IL-10 inducing peptides if MHC II binding status of the query peptide is not known, as we have not used MHC II non-binders in our dataset. Thus it is possible that our model may predict a MHC II non-binder as IL-10 inducing peptide. In order to overcome this problem, we also developed models using the alternate dataset to discriminate IL-10 inducing and MHC II non-binders. We tested two of the machine learning methods – SVM and Random Forest that showed the best results on the dataset of IL-10-inducing MHC II binders and IL-10 non-inducing MHC II binders. We used 80% of the data for training and testing our models using five-fold cross validation technique. The remaining 20% data called independent dataset was used for external validation of our models. Our best SVM model achieved an accuracy of 76.44% with the MCC of 0.54, when evaluated using five-fold cross-validation. We also tested the performance of this model on the independent dataset and achieved an accuracy of 75.93% with MCC of 0.54. The Random Forest-based method showed a similar performance with 76.33% accuracy and 0.53 MCC, when tested using five-fold cross validation. The performance of the above model on the independent dataset was 77.31% accuracy and 0.58 MCC.

### Service to the scientific community

One of the major goals of our group is to provide service to the community based on research carried out in our group. Thus, we developed a user-friendly webserver that integrates models developed in this study. The web-interface developed for the users predicts a query peptide to be IL-10 inducer or non-inducer based on the prediction models developed on the dataset containing IL-10-inducing MHC II binding peptides as positives and IL-10 non-inducing MHC II binders as negatives. However, such a model can falsely predict an MHC II non-binder to be an IL-10-inducing peptide. Thus, we developed separate prediction models that distinguish IL-10-inducing MHC II binders from MHC II non-binders. The web-interface designates a query peptide to be IL-10-inducing only if it is predicted to be positive by both of the above-mentioned models.

The web interface of the server has three main modules; i) Predict, ii) Design and iii) Protein Scan. The ‘Predict’ tool allows a user to identify IL-10 inducing peptides in a given library of peptides. The ‘Design’ module facilitates the user to generate all possible analogs of the query peptide and identify the best analogs for inducing cytokine IL-10. The ‘Protein Scan’ module was developed for scanning IL-10 inducing regions in a query protein. Our web server has been designed using a responsive HTML template for adjusting to the browsing device. Thus, our webserver is compatible with a wide range of devices including the desktops, tablets and smartphones.

**Figure 5:**
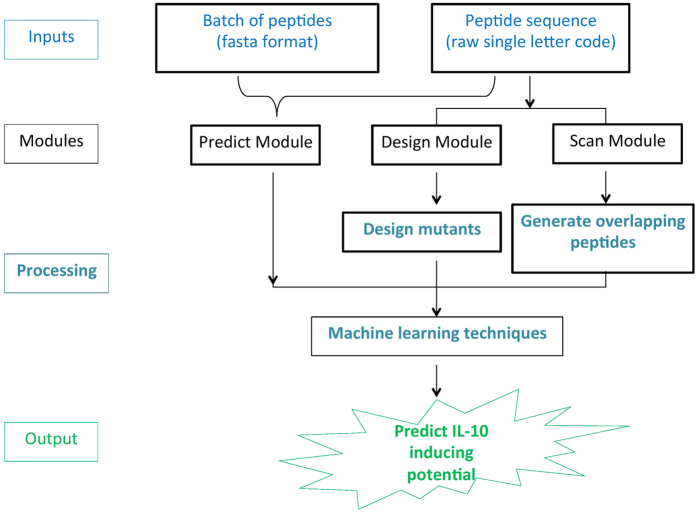
Flow chart shows processing of data in android based mobile app, developed for predicting IL-10 inducing peptides.

In addition to the webserver, we also developed a standalone version of IL-10pred using wxPython. Keeping in view the exponential growth of usage of smart phone users in last decade, we also developed an Android-based mobile app using the Kivy package. The workflow of the IL-10 mobile app has been summarized in the Fig. 5. All these applications are accessible at the URL http://crdd.osdd.net/raghava/IL-10pred/.

## Discussion

Immunosuppression is a systemic response that may be desired in some cases like asthma therapy and inappropriate in some other conditions like cancer. Peptide-based immunotherapy has been shown to be capable of capitalizing on both of these flip sides by removal or introduction of IL-10 inducing epitopes in the antigen. In an attempt to develop a therapy for asthma treatment, the IL-10 inducing epitopes were shown to suppress the immune response evoked by other epitopes of the same antigen^45^. On the other hand, removal of IL-10 inducing T cell epitopes from the insulin-like growth factor-binding protein 2 (IGFBP2) vaccine conferred potent anti-tumor activity^46^. With an increased understanding of IL-10 inducing epitopes, their inclusion or exclusion becomes an important consideration in a vaccine design.

In the present study, we have made a systematic attempt to understand the nature of IL-10 inducing peptides and to develop models for predicting IL-10 inducing peptides. This is the first *in silico* study on IL-10 peptides though there is limited information available in the literature. In order to perform this type of study, one needs to have a dataset of inducing and non-inducing peptides. Thus, we examined the experimentally validated MHC class-II binders in IEDB database^47^ and extracted IL-10 inducing and non-inducing MHC class-II binders. The dataset of experimentally validated IL-10 inducing and non-inducing peptides is the backbone of this study. We analyzed these peptides to understand compositional and positional preferences of residues in IL-10 inducing peptides using Two-Sample Logo and compositional analysis. As shown in the Results section, certain types of residues are more abundant in IL-10 inducing peptides. In addition, positional preferences of certain types of residues were also observed in the IL-10 inducing peptides. This indicates that IL-10 inducing and non-inducing peptides differ in terms of residue composition. Thus composition can be used to discriminate these two types of peptides.

**Figure 6:**
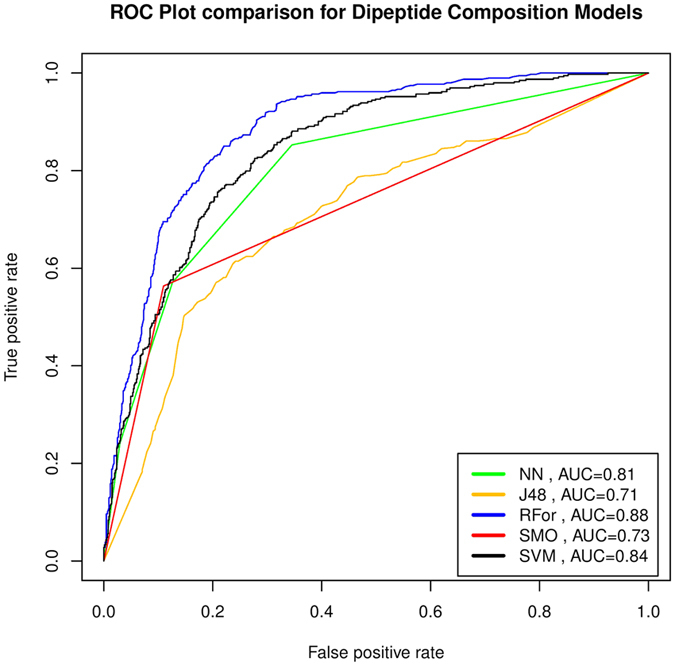
ROC plot shows performance of dipeptide composition based models developed using different machine learning techniques; Random Forest (RFor) based model achieves maximum AUC 0.88.

We tried a wide range of classifiers to build models for predicting IL-10 inducing peptides. Further, we also used a wide range of features particularly compositional features for discriminating IL-10 inducing and non-inducing peptides. As anticipated, models based on compositional features particularly based on DPC, classify IL-10 inducing and non-inducing peptides with high performance. Initially, SVM-based models were developed using different sequence features and achieved reasonably good performances. We also tried popular classifiers available in the software package WEKA and achieved moderate performances using different classifiers. Our Random Forest-based model developed using DPC attained the highest performance among all the classifiers used in the present study (Fig. 6).

## Conclusion

In a scenario where direct use of IL-10 as a therapeutic model has revealed toxic effects, peptide-based epitopes that induce IL-10 provide a promising alternative. It has been shown in previous studies that blocking the IL-10 receptor using antibodies could enhance the efficiency of subunit vaccines, for example, in the case of mycobacteria^48^,^49^. Thus, blocking the IL-10 induced immunosuppression could be an important aspect of subunit vaccine design. Although numerous methods are available for *in silico* prediction of T cell epitopes^33^, computational methods are not available for predicting IL-10 inducing epitopes. The present work is an attempt to provide a platform for addressing this important aspect. In order to facilitate the scientific community in developing better methods for prediction of IL-10 inducing peptides, we have provided our datasets used in the present study.

## Methods

### Building Dataset

One of the major challenges for this type of work is to create an authentic dataset containing experimentally validated IL-10 inducing and non-inducing peptides. In this study, the dataset is derived from the IEDB database^47^, which is the largest repository of immune epitopes. The MHC class II binders that were reported to trigger IL-10 release were extracted from the IEDB. We extracted experimentally validated MHC class II binders that elicit cytokine IL-10; these peptides were assigned as IL-10 inducing peptides. We also extracted MHC class II binders reported not to trigger IL-10 release from IEDB. We assigned these MHC class II binding peptides as non-inducing peptides. In order to remove redundancy, we removed identical peptides from both, IL-10 inducing and non-inducing peptides. Our final dataset called the main dataset consists of 394 IL-10 inducing and 848 non-inducing peptide sequences enlisted in Table S4, with unique positive and negative sequences.

In addition to the main dataset, we also created another dataset called the alternate dataset (sequences provided in Table S5). This dataset contains different negative instances than in the main dataset. This dataset contains MHC II non-binders as negative instances instead of MHC II binders. The alternate dataset contains 461 IL-10-inducing MHC II binders as positive instances. In order to create a dataset of negative instances, we extracted 621 MHC II non-binders from the MHCBN database^50^. In summary, our alternate dataset consists of 461 IL-10 inducing peptides and 621 MHC II non-binders. We built this dataset to classify IL-10 inducers and MHC II non-binders.

### Computation of the Residue Composition

In the past, compositional features of the peptide sequences have been used successfully for developing methods for predicting the function of peptides^43^,^51^. Thus in this study also models have been developed using different types of composition that includes amino acid and dipeptide composition. The composition features (AAC and DPC) were calculated using the in-house Perl scripts based on the following equations 1 and 2.


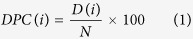



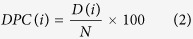


In the above equations, **AAC(i)** is the percent amino acid or residue composition of the residue type **i**. **R(i)** is the number of residues type **i** and **N** is the total number of residues in a peptide sequence. **DPC(i)** is the percent of dipeptide composition for residue type **i**. **D**(**i**) is the number of dipeptides of type **i** and N is the total number of dipeptides in a peptide sequence.

### Binary Profile

It is another important feature for representing peptide sequences. In the case of binary profile, each of the 20 types of natural amino acid is represented as binary vectors of dimension twenty (e.g. Ala by 1,0,0,0,0,0,0,0,0,0,0,0,0,0,0,0,0,0,0,0; Cys by 0,1,0,0,0,0,0,0,0,0,0,0,0,0,0,0,0,0,0,0). The sequence length in the positive and the negative datasets is variable, but the input vector for applying the machine learning techniques should be of fixed length. Since the minimum length of the sequences is 8 for both the positive and the negative sequences, substrings of length 8 were taken from the N-terminus as well as C-terminus of each sequence and concatenated to have derived sequences of fixed length (16) for each of original sequences. Such derived sequences were used to generate the binary profile.

### Two-sample logo

The sequences derived for obtaining the binary profile were also used for generating a Two-Sample logo (TSL)^52^ using the web tool available at http://www.twosamplelogo.org/cgi-bin/tsl/tsl.cgi, since this tool also requires a fixed length input sequence criterion. Since the minimum length of the peptides in the dataset was 8 amino acids, the TSL consists of 8 residue positions from each of the N and C termini leading to a profile of 16 residue positions.

### Machine-learning Techniques

The Support Vector Machine (SVM)-based prediction models were developed using the package SVM^*light *^53. The radial basis function kernel was mainly used in this study; different parameters were optimized to get the best performance on the training dataset. In addition, some commonly used classifiers were also used for developing prediction models. These classifiers (e.g., Random Forest, IBK, SMO and J48) were implemented using the WEKA package^54^.

### Feature Selection

In this study, we also used the WEKA^54^ package for selecting important features from different compositional features. We used CFSubSetEval algorithm with default parameters for the selection of significantly relevant features. These selected features were examined to understand nature of IL-10 inducing peptides as well as for developing the prediction models (Table S3).

### Cross-validation

In order to train, test and evaluate our models, we used the five-fold cross validation technique. This is a standard technique, commonly used in this type of studies; details are available in the previous studies^51^. In summary, the whole dataset is divided into five equal parts, with all five sets having an equal number of positive and negative instances. The four sets are used for training, while the remaining set is used for testing. This process is iterated five times so that each set is used for testing.

### Evaluation parameters

Model evaluation is an important step to estimate the efficiency of the model. We have used well-established evaluation parameters that include sensitivity, specificity, accuracy and MCC.


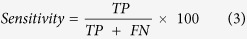



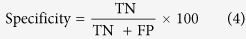



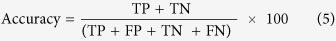



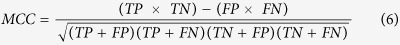


TP =True Positive, FP =False Positive, TN =True Negative, FN =False Negative.

## Additional Information

**How to cite this article:** Nagpal, G. *et al*. Computer-aided designing of immunosuppressive peptides based on IL-10 inducing potential. *Sci. Rep.* **7**, 42851; doi: 10.1038/srep42851 (2017).

**Publisher's note:** Springer Nature remains neutral with regard to jurisdictional claims in published maps and institutional affiliations.

## Supplementary Material

Supplementary Tables

## Figures and Tables

**Figure 1:**
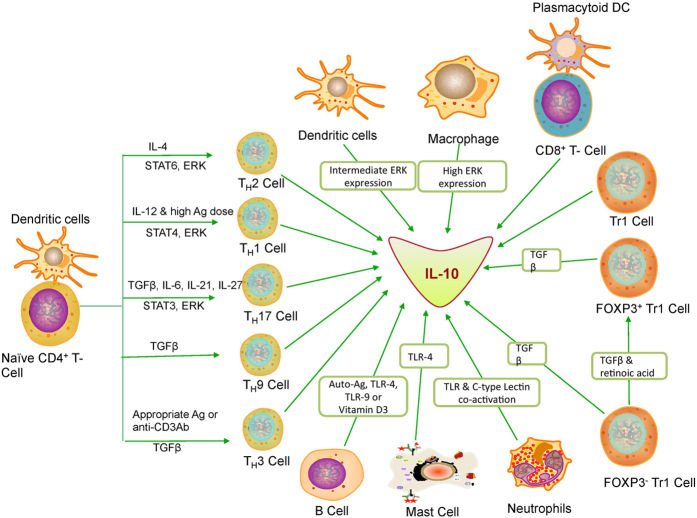
Role of different types of immune cells in production of interleukin-10.

**Figure 2:**
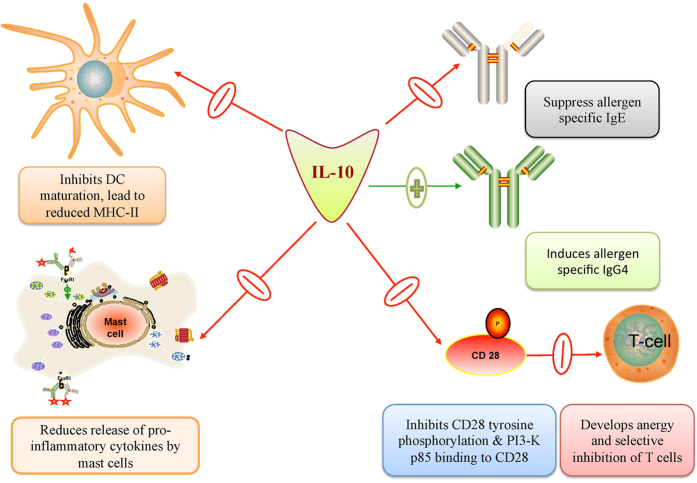
A schematic diagram of immunosuppressive mechanism of Interleukin-10. It mainly involves dendritic cells (DC), major histocompatibility complex (MHC), phosphatidylinositol 3-kinase (PI3-K) and immunoglobulin.

**Table 1:** Exclusive motifs found in IL-10 inducing and non-inducing peptides; motifs searched using MERCI program.

IL-10 inducing peptide				IL-10 Non-inducing peptides			
Motif	# of sequences	Coverage of positive dataset	# of unique Sequences	Motif	# of sequences	Coverage of negative dataset	# of unique Sequences
R-D-H	12	12	12	A-T-A-A-T	32	32	32
L-A-E-Y	11	23	11	V-W-Q	26	58	26
I-F-L-V	10	33	10	PG-P-G	25	83	25
G-A-Q-G-K	10	43	10	K-P-G-D	22	104	21
H-F-T	10	52	9	KDV	21	124	20
E-V-C-G	10	61	9	A-G-A-T-A	27	143	19
R-L-K-V-A	10	69	8	V-GP	25	163	20
PLL	9	78	9	EA-A-T	24	181	18
I-K-R-K	9	87	9	A-VA-V	23	199	18
E-R-V-V	9	95	8	VP-K	23	217	18

**Table 2:** The performance of SVM based models developed using different peptide features.

Features	Threshold	Sensitivity	Specificity	Accuracy	MCC
Whole peptide length					
AAC	−0.5	70.05	73.35	72.30	0.41
DPC	−0.3	79.95	77.71	78.42	0.55
split-AAC	−0.6	70.05	75.35	73.67	0.43
split-DPC	−0.4	67.77	75.00	72.71	0.41
NT8					
AAC	0.3	63.20	63.56	63.45	0.25
DPC	−0.4	67.01	66.63	66.75	0.32
Binary	−0.2	64.72	68.28	67.15	0.31
CT8					
AAC	−0.2	62.69	64.39	63.85	0.25
DPC	−0.4	67.77	64.03	65.22	0.30
Binary	−0.3	63.20	62.74	62.88	0.24
NT8CT8					
AAC	−0.5	70.05	69.46	69.65	0.37
DPC	−0.3	77.92	78.42	78.26	0.54
Binary	−0.4	68.27	64.03	65.38	0.30

**Table 3:** The performance of models based on different classifiers developed using amino acid and dipeptide composition; classifiers implemented using WEKA.

Classifier	Threshold	Sensitivity	Specificity	Accuracy	MCC	Parameters
Amino Acid Composition (AAC)						
IBK	0.3	71.83	74.29	73.51	0.44	-K 6
SMO	0.5	44.42	88.33	74.40	0.37	-C 5 –G 0.001
J48	0.2	66.50	69.10	68.28	0.34	-C 0.4 -M 9
Random forest	0.3	80.46	79.95	80.11	0.58	-I 300
Dipeptide Composition (DPC)						
IBK	0.2	76.40	76.18	76.25	0.50	-K 3
SMO	0.5	56.35	89.03	78.66	0.49	-C 5 –G 0.001
J48	0.1	67.26	67.10	67.15	0.32	-C 0.4 -M 2
Random forest	0.3	79.70	81.96	81.24	0.59	-I 600

**Table 4:** The performance of models based on WEKA classifiers developed using with selected features obtain from amino acid and dipeptides composition.

Classifier	Threshold	Sensitivity	Specificity	Accuracy	MCC	Parameters
16 selected features from amino acid composition						
IBK	0.3	70.30	70.99	70.77	0.39	-K 6
SMO	0.5	37.60	88.92	72.46	0.31	-C 5 –G 0.001
J48	0.2	66.50	69.10	68.28	0.34	-C 0.3 -M 9
Random forest	0.3	79.95	78.42	78.90	0.55	-I 700
57 selected features from dipeptide composition						
IBK	0.2	72.84	71.58	71.98	0.42	-K 1
SMO	0.5	46.70	87.38	74.48	0.37	-C 5 –G 0.01
J48	0.3	72.84	70.52	71.26	0.41	-C 0.4 -M 2
Random forest	0.3	77.66	77.00	77.21	0.52	-I 200
